# Normospermic Patients Infected With *Ureaplasma parvum:* Role of Dysregulated miR-122-5p, miR-34c-5, and miR-141-3p

**DOI:** 10.20411/pai.v8i2.603

**Published:** 2024-01-05

**Authors:** Marilena Galdiero, Carolo Trotta, Maria Teresa Schettino, Luigi Cirillo, Francesca Paola Sasso, Francesco Petrillo, Arianna Petrillo

**Affiliations:** 1 Department of Experimental Medicine, Section of Microbiology and Virology, University of Campania Luigi Vanvitelli, 80138, Naples, Italy. Department of Experimental Medicine, Section of Pharmacology, University of Campania Luigi Vanvitelli, 80138, Naples, Italy; 2 Department of Gynecology and Obstetrics University of Campania Luigi Vanvitelli Naples Italy; 3 Department of Neurosciences, Reproductive Sciences and Odontostomatology, University of Naples Federico II, Naples Italy; 4 Department of Dermatology and Venereology. University of Rome La Sapienza Italy; 5 Department of Pediatrics University of Milan, Italy

**Keywords:** *Ureaplasma parvum*, miRNA, sperm motility

## Abstract

**Background:**

*Ureaplasma parvum* (UP) is a causative agent of non-gonococcal urethritis, involved in the pathogenesis of prostatitis and epididymitis, and it could impair human fertility. Although UP infection is a frequent cause of male infertility the study evidence assessing their prevalence and the association in patients with infertility is still scarce. The molecular processes leading to defects in spermatozoa quality are not completely investigated. MicroRNAs (miRNAs) have been extensively reported as gene regulatory molecules on post-transcriptional levels involved in various biological processes such as gametogenesis, embryogenesis, and the quality of sperm, oocyte, and embryos.

**Methods:**

Therefore, the study design was to demonstrate that miRNAs in body fluids like sperm could be utilized as non-invasive diagnostic biomarkers for pathological and physiological conditions such as infertility. A post-hoc bioinformatics analysis was carried out to predict the pathways modulated by the miRNAs dysregulated in the differently motile spermatozoa.

**Results:**

Here it is shown that normospermic patients infected by UP had spermatozoa with increased quantity of superoxide anions, reduced expression of miR-122-5p, miR-34c-5, and increased miR-141-3p compared with non-infected normospermic patients. This corresponded to a reduction of sperm motility in normospermic infected patients compared with normospermic non-infected ones. A target gene prediction presumed that an essential role of these miRNAs resided in the regulation of lipid kinase activity, accounting for the changes in the constitution of spermatozoa membrane lipids caused by UP.

**Conclusions:**

Altogether, the data underline the influence of UP on epigenetic mechanisms regulating spermatozoa motility.

## INTRODUCTION

Recent data from the World Health Organization (WHO) revealed that, globally, about 48 million couples and 186 million individuals have infertility (World Health Organization. Laboratory manual for the examination and processing of human semen. WHO, Geneva, 2021). Many factors contribute to human infertility, in which genital tract infections are represented as one of the major factors with a prevalence of 6%–10% worldwide [1]. Several studies revealed that *Chlamydia trachomatis, Mycoplasma spp*, and *Ureaplasma spp* play an important role in both genital infections and infertility representing the main agents encountered in male infertility cases. Indeed, in these *in vitro* studies the authors demonstrated that the co-incubation of sperm with Chlamydia caused a significant drop in the number of motile sperm and premature sperm death. Eley et al measured lipopolysaccharide levels in sperm and correlated them with parameters of sperm quality, including that of sperm function. It has also been shown that lipopolysaccharide induces excessive production of reactive oxygen species (ROS) in spermatozoa and that this reduces sperm motility [2–4]. Despite the fact that other pathogens such as the bacteria *Chlamydia trachomatis* (CT) and *Neisseria gonorrhea* (NG), molds, and trichinosis can cause genital tract infections [5], even if at relatively low incidence rates, *U. urealyticum*, a natural resident of the male urethra, is the main causative organism of male genital tract infections contaminating seminal fluid during ejaculation and contributing to both genital infections and male infertility. Infections caused by these microorganisms are often asymptomatic, hence the exact role and mechanisms by which genitourinary microorganisms affect human fertility potential remain unknown [3, 6]. Friberg and Gnarpe were the first to prove the high frequency of Ureaplasma strains in the sperms of infertile men (76%), compared to fertile men (19%) [7]. Thereafter, the correlation between Ureaplasma spp infections and male infertility were extensively studied. To date, the frequency of isolated Ureaplasma strains in seminal fluids of infertile men ranged from 5% to 58%, in contrast to 3%–31% of fertile men [3, 8]. In addition, Fowlkes et al showed that the semen parameters of men with Ureaplasma infection were altered, compromising sperm function [9]. Ureaplasma spp naturally colonize the male urethra and consist of 2 species, *Ureaplasma parvum* (UP) and *Ureaplasma urealyticum* (UU) [10]. Firstly, Zinzendorf et al in a study reported the effect of U. urealyticum infection on semen analyzing parameters in male patients with infertility [11] and compared it with data from normal patients. Other studies are consistent with this, researchers have shown that U. urealyticum infection in the male genital tract negatively affects semen quality [12] significantly involving the pH value, liquefaction time, concentration, and motility in semen samples. Most studies examined the role of UU in male infertility while little evidence about the mechanisms of male infertility induced by UP infections has been reported [13–15]. Since the 2 species differ in pathogenicity, the impact of UP on sperm function could be different [9].

Therefore, understanding the primal molecular processes in response to UP infections could be relevant for the selection of innovative therapeutic and diagnostic targets. In this context, recent studies reminded us that several miRNAs showed a dynamic expression pattern during the life and maturation of spermatozoa [16]. The microRNAs (miRNAs) are small non-coding RNA molecules, tissue-specific, implicated in the posttranscriptional regulation of gene expression and constitute a kind of molecular marker for tissue and body fluids identification [16, 17]. These molecules play regulatory roles in various biological processes, including development, cell proliferation, cell differentiation, cell death, metabolic control, transposon silencing, and anti-viral defense. Several studies have shown that sperm can be distinguished from other body fluids using the assessment of specific miRNAs evaluated by RT-PCR [18, 19]. The motility of spermatozoa is an important parameter for evaluating semen quality and an essential factor for fertilization events. Therefore, a seminal marker that could provide information about the spermatogenesis status during infection will be of immeasurable value for diagnostic teams [20, 21]. Different miRNAs involved in the regulation of sperm motility have been identified, such as hsa-miR-141-3p, hsa-miR-122-5p, and hsa-miR-34c-5p [19, 22–26].

However, no evidence on the role of these miRNAs in UP infections and their relationship with semen quality has been reported. Therefore, the present study aimed to examine the level of expression of hsa-miR-141-3p, hsa-miR-122-5p, and hsa-miR-34c-5p in seminal fluids, obtained from patients infected by UP. In addition, the prediction of target genes regulated by the tested miRNAs could contribute to the understanding of the molecular mechanism underlying the infertility caused by UP [20, 23, 24].

## METHODS

### Collection and Analysis of Semen Samples

Our study was conducted on 52 semen samples, collected, and analyzed at the Andrology clinic, University of Campania Luigi Vanvitelli (Vanvitelli University) in Naples between July and October 2022. Written informed consent was obtained to authorize the anonymous collection of sperm analysis results and exuberant sperm samples for research aims according to the Ethical Committee of the Vanvitelli University (Protocol number 0022538/I, 20/07/2022). The ejaculates were collected from patients attending the clinical center with ages between 25 and 39 years old.

Seminal fluids were collected by masturbation after 3 to 5 days of recommended sexual abstinence. Before collecting the samples, participants were recommended to wash their hands and genital area with water and soap.

Samples were collected in sterile plastic containers and transported to the microbiology laboratory within 1 hour. They were placed in the incubator for 30 minutes for liquefaction and subjected to routine semen analysis and polymerase chain reaction (PCR) to detect bacteria. Sperm concentration, vitality, and motility were evaluated, using the SQA-V GOLD method (Ab analitica, Padova, Italy), in agreement with the WHO guidelines 2010 [21, 27].

### DNA Extraction and UP Detection

The extraction of genomic DNA is a necessary step before the analysis of clinical samples with the Anyplex™ II STI-7 Detection Kit. The extraction was performed with GeneAllxgene Cell SV mini-Kit (GeneAll Biotechnology, Seoul, South Korea) according to the manufacturer's instructions. UP was detected by Anyplex II STI-7 (Seegene, Seoul, Korea), in agreement with the manufacturer's instructions [28].

Reference testing was performed using the multiplex real-time PCR Anyplex™ II STI-7 Detection Kit. Briefly, for each sample, 5 µL of the extracted DNA was added to a final volume of PCR Master Mix consisting of 5 µL of 4X STI-7 TOM (amplification and detection reagents), 5 µL of 4X Anyplex PCR Master Mix (DNA polymerase, uracil-DNA glycosylase, and buffer containing dNTPs), and 5 µL of RNase-free water (ultrapure quality, PCR grade). One positive and one negative control were included in each run. The CFX96Ô Real-Time PCR system (Bio-Rad) was set up to carry out the DNA amplification and detection. The real-time PCR was programmed according to the guiding procedures on the manufacturer's protocol and was examined with Seegene viewer software.

### Randomization of Semen Samples

According to reference values for sperm concentration and UP presence, semen samples were selected, anonymized, and randomized as follows (n= 10 per group): i) (N): sperm concentration > 15 M/mL and absence of bacteria; ii) (I): sperm concentration > 15 M/mL and presence of UP.

### Sperm miRNA Isolation

Within a few hours after collection, sperm cells were isolated from the exuberant human seminal plasma according to the Ethical Committee of Vanvitelli University (Protocol number 0022538/I, 20/07/2022). Specimens were centrifuged at 500*g* for 10 minutes at room temperature; then sperm pellets were stored at -80°C in a Frozen Storage Buffer (FBS) until a short-term usage. Then, sperm pellets were thawed, washed twice with phosphate-buffered saline (PBS) to remove FSB, suspended in a Somatic Cell Lysis Buffer and incubated on ice for 30 minutes to obtain the somatic cell lysis [27, 29]. The presence of somatic cells was verified with microscopic examination and the process was repeated until somatic cells were no longer visible [27].

Total RNA was isolated by using miRNeasy Mini kit (Qiagen, USA), according to the manufacturer's protocol (Purification of Total RNA, Including Small RNAs, from Animal Cells) with some modifications during the lysis stage, as previously described [22, 23]. Briefly, frozen sperm pellets were homogenized in the lysis buffer for 90 seconds at low settings. After incubation at 65°C for 5 minutes, microscopic examination was used to verify complete lysis of sperms. When this was achieved, the default protocol was resumed. After homogenization, Syn-cel-miR-39 was added to each preparation before RNA purification on spin columns, to monitor RNA isolation efficacy. Then, chloroform was added before centrifugation, to obtain the separation of the upper aqueous colorless phase containing RNA from the other 2 phases (the white interphase and the lower red organic phase). Then, RNA samples were purified by spin columns, before RNA elution with RNAse-free water. A NanoDrop™ 1000 (Thermo Scientific) spectrophotometer was used to evaluate RNA concentration and purity.

### miRNA qRT-PCR

Mature miRNAs were converted to cDNA by using MiScript II Reverse Transcription Kit (Qiagen) and GeneAmp PCR System 9700 (Applied Biosystems). After the addition of the buffer, nucleic mix, reverse transcriptase mix, RNAse-free water, and RNA, samples were incubated for 60 minutes at 37°C and for 5 minutes at 95°C, according to the manufacturer's protocol. Then, CFX96 Real-Time System C1000 Touch Thermal Cycler (BioRad Laboratories, Inc), miScript SYBR Green PCR kit (Qiagen) and specific miScript primer Assays (MS00003507, MS00003416, MS00003332–Qiagen, USA) were used to evaluate hsa-miR141-3p (Accession number MIMAT0000432), hsa-miR-122-5p (Accession number MIMAT0000421), and hsa-miR-34c-5p (Accession number MIMAT0000686) expression levels by triplicate measurements of each sample. An initial incubation step at 95°C for 15 minutes was performed to activate the DNA polymerase, then the cycling conditions for qPCR were 15 seconds at 94°C (denaturation), 30 seconds (annealing) at 55°C, 30 seconds at 70°C (extension). These steps were repeated for 40 cycles. Then, miRNA levels were normalized by using the combination of 2 internal controls, hsamiR-100-5p (MS00031234 Qiagen; Accession number MIMAT0000098) and hsa-miR-30a-5p (MS00007350 Qiagen; Accession number MIMAT0000087), which was recently found to result in the best normalization strategy for miRNA quantification in sperm cells [30]. The amplification of Ce_miR-39-3p (MS00019789 Qiagen; Accession number MIMAT0000010) was a positive control for efficient RNA isolation. The 2^-ΔΔCt^ method was used for miRNA level quantification, with Ct values detected by CFX ManagerTM Software (BioRad Laboratories, Inc) [31].

### Superoxide Anion Assay

Superoxide anion content was determined in 1 x 10^6^ spermatozoa for each group, by using the General Superoxide Anion Assay Kit (MBS8305395 My BioSource), according to the manufacturer's protocol. Briefly, 20 µL of each sample was transferred to a 96-well plate and incubated with the substrate (20 µL) at 37°C for 20 minutes. After incubation, reagents I (80 µL, dissolved in distilled water) and II (80 µL, dissolved in alcohol) were added in succession to the test samples. Color intensity, linear to the Superoxide Anion content in the sample, was measured at 530 nm using a microplate reader (TECAN, Männedorf, Swiss). The superoxide anion amount was obtained referring to the standard curve supplied by the kit.

### miRNA Target Gene Prediction

**Figure 1.**
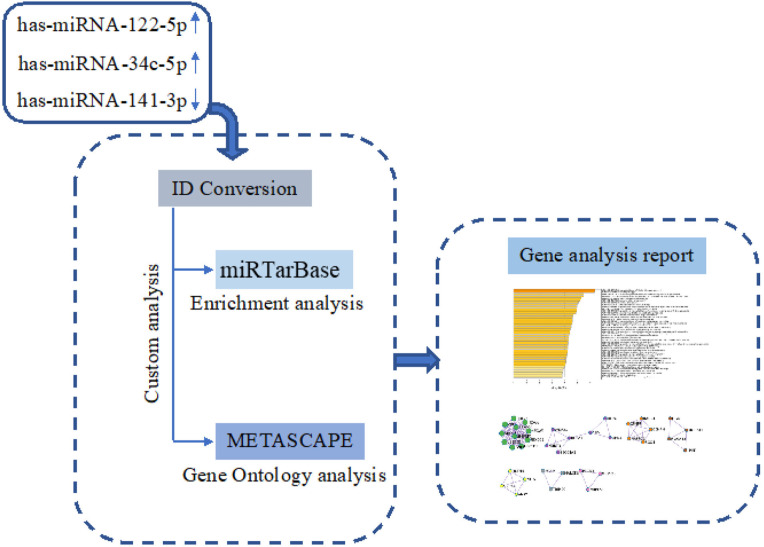
Schematic workflow of target genes prediction process.

The prediction of hsa-miR-122-5p, hsa-miR-34c-5p, and hsa-miR-141-3p target genes was achieved using miRTarBase software (miRTarBase v8.0 beta, Hsinchu, Taiwan). According to the set parameter (*P* value < 0.05), the potential target genes commonly regulated were selected. Metascape software (Metascape v5.0, San Diego, CA, USA) was used for the enrichment of gene ontology (GO) and the identification of protein-protein interaction networks (PPI-Nets) [17] (Figure 1).

### Statistical Analysis

Student's *t* test was used for data analysis. The association between sperm miRNA levels and sperm variables (bacterial load UP, sperm concentration, and motility) was evaluated by Pearson correlation analysis. A *P* value < 0.05 was considered significant. Statistical analysis was performed using GraphPad Prism 6.0 software [32–34].

## RESULTS

### Analysis of Sperm Motility

Total sperm motility was significantly reduced in the I group compared to N (fold= -1.54, *P* < 0.05 vs N), with a parallel increase in not-motile sperms (fold = +1.44, *P* < 0.05 vs N). Moreover, when analyzing progressively motile and non-progressively motile sperms, a decrement of progressive sperms was noted when comparing the I and N groups (fold = -1.57, *P* < 0.05 vs N), paralleled by a marked increase of non-progressive sperms (fold = +3.6, *P* < 0.05 vs N) (Table 1). Immobile sperms increased up to 1.6-fold, *P* <0.05 comparing the N group against I (Table 1).

**Table 1.** Characteristics of the Total Population

	Total motility	PR (%)	NP (%)	IM (%)
**N (n=10)**	52 ± 6.0	92 ± 6.3	8.0 ± 2.7	**48 ± 4.0**
**I (n=10)**	33 ± 5.0*	64 ± 5.0*	36 ± 2.0*	67 ± 3.0*

Data are reported as mean ± S.D. (n=10 per group).

N= non-infected samples; I= infected samples; PR= progressive motility of spermatozoa; NP= non-progressive motility of spermatozoa; IM= immotile spermatozoa;

* *P* < 0.05 vs N.

**Figure 2.**
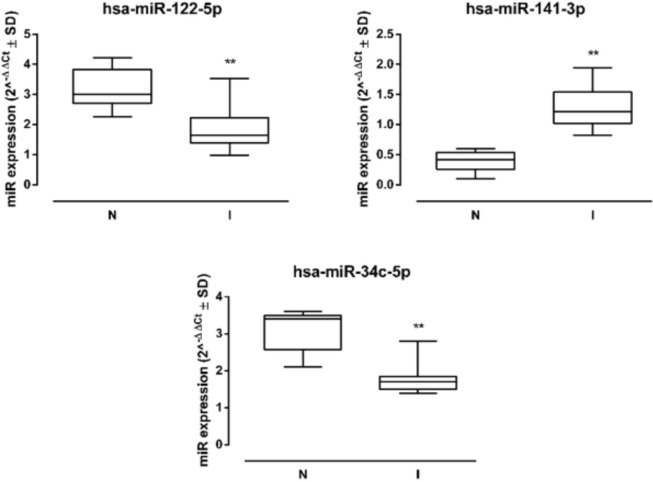
**Sperm miRNA levels.** Human sperm hsa-miR-122-5p, hsa-miR-141-3p, and hsa-miR-34c-5p levels in non-infected (N) and infected (I) samples (N=10 per group), and miRNA expression levels are reported as 2^-ΔΔCt^ ± SD (box plots). ** *P* < 0.01 vs N.

The 3 miRNAs analyzed showed a significant dysregulation in samples from UP-infected patients. In particular, miR-141-3p was significantly increased in I sperms (fold =+1.71, *P* < 0.05 vs N) compared to N samples, while miR-122-5p and miR-34c-5p were significantly reduced (miR-122-5p fold = -1.96, *P* < 0.01; miR-34c-5p fold = -1.94, *P* < 0.01 vs N) (Figure 2).

### Relationship Between Semen UP Infection and Sperm miRNA Levels

The correlation analysis between UP bacterial load and sperm miRNA expression evidenced a positive correlation between UP with miR-141-3p *(r* = 0.574, *P* = 0.003), and a negative association with miR-122-5p (r = -0.553, *P* = 0.004) and miRr-34c-5p (r = -0.540, *P* = 0.005) (Table 2).

**Table 2.** Association Between Semen UP and Sperm miRNAs

	miR-122-5p (2^-ΔΔCt^)	miR-141-3p (2^-ΔΔCt^)	miR-34c-5p (2^-ΔΔCt^)
**P (++) 10** ^5^	(*r*) -0.553**	(*r*) 0.574**	(*r*) -0.540**

Pearson's coefficient (*r*) between the presence of UP and miRNAs in sperm samples.

** *P* < 0.01.

### Correlation Between miRNAs and Sperm Motility in UP Infection

Levels of miR-141-3p were associated with a reduction of sperms exhibiting a forward progression (r = -0.617, *P* = 0.002). Indeed, miR-141-3p expression was associated with a higher percentage of not-motile (r = +0.582, *P*=0.004) and non-progressive sperm cells (r = +0.719, *P* = 0.002). Overall, higher miR-141-3p levels indicated a significant reduction in total sperm motility (r = -0.664, *P* = 0.003). Conversely, miR-122-5p and miR-34c-5p levels correlated with an increase of total sperm motility (r = +0.730, *P* = 0.001; r = +0.676, *P* = 0.005, respectively) and progressive spermatozoa (r = +0.614, *P* = 0.002, r = 0.605, *P* = 0.003, respectively). These 2 miRNAs were associated with the reduction of non-progressive motility (r = -0.648, *P* = 0.01, r = -0.592, *P* = 0.02, respectively) and not-motile sperm cells (r = -0.619, *P* = 0.002; r = -0.575, *P* = 0.005, respectively) (Table 3).

**Table 3.** Association Between miRNAs and Sperm Characteristics

Hsa-miRs	Total motility (PR+NP)	PR	NP	IM
**122-5p**	(*r*) 0.730**	(*r*) 0.614**	(*r*) -0.648*	(*r*) -0.619**
**141-3p**	(*r*) -0.664**	(*r*) -0.617**	(*r*) 0.719**	(*r*) 0.582**
**34c-5p**	(*r*) 0.676**	(*r*) 0.605**	(*r*) -0.592*	(*r*) -0.575**

Pearson's coefficient (*r*) for the relation of miRNA levels with spermatozoa motility in UP-infected samples.

PR= progressive motility of spermatozoa; NP= non-progressive motility of spermatozoa; IM= immotile spermatozoa;

* *P* < 0.05 and

** *P* < 0.01.

### Superoxide Anion Level in UP Infection

**Figure 3.**
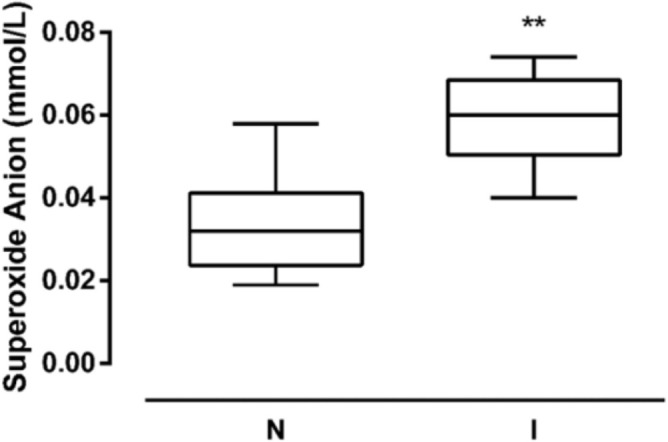
**Sperm superoxide anion levels.** Superoxide anion content in spermatozoa from non-infected (N) and infected (I) samples (N=26 per group). Data are reported as mmol/L ± S.D. ** *P* < 0.01 vs N.

The superoxide anion (O-2) levels were evaluated to determine the oxidative stress damage caused by UP infection. The O-2 concentration was 0.035 and 0.062 mmol/L, in N and NI samples, respectively. The O-2 levels were significantly elevated in I sperms compared to the N group, showing a 77.1% increase (*P*<0.01) (Figure 3).

### Correlation Between Superoxide Anion Levels, UP Bacterial Load, miRNAs and Sperm Motility

Pearson correlation analysis showed a positive association between O-2 content in sperm samples and UP infection (r = 0.727; *P* = 0.001). Moreover, O-2 levels were negatively correlated with both sperm miR-122-5p and miR-34c-5p (r = -0.780, *P* = 0.002 and r = -0.775, *P* = 0.002 respectively), while they showed a positive association with miR-141-3p sperm levels (r = 0.775; *P* = 0.003). In addition, O-2 was negatively correlated with sperm total motility (r = -0.793; *P* = 0.001) (Table 4).

**Table 4.** Association Between Superoxide Anion Levels, UP, miRNAs, and Sperm Motility

	UP (++) 10^5^	miR-122-5p	miR-141-3p	miR-34c-5p	Total motility
**Superoxide Anion**	(*r*) 0.727**	(*r*) -0.780**	(*r*) -0.580**	(*r*) -0.775**	(*r*) -0.793**

Pearson's coefficient (*r*) for the relation of superoxide anion levels with UP infection, miRNA levels and total motility in sperm samples.

** *P* < 0.01.

### Identification and Characterization of Target Genes

**Figure 4.**
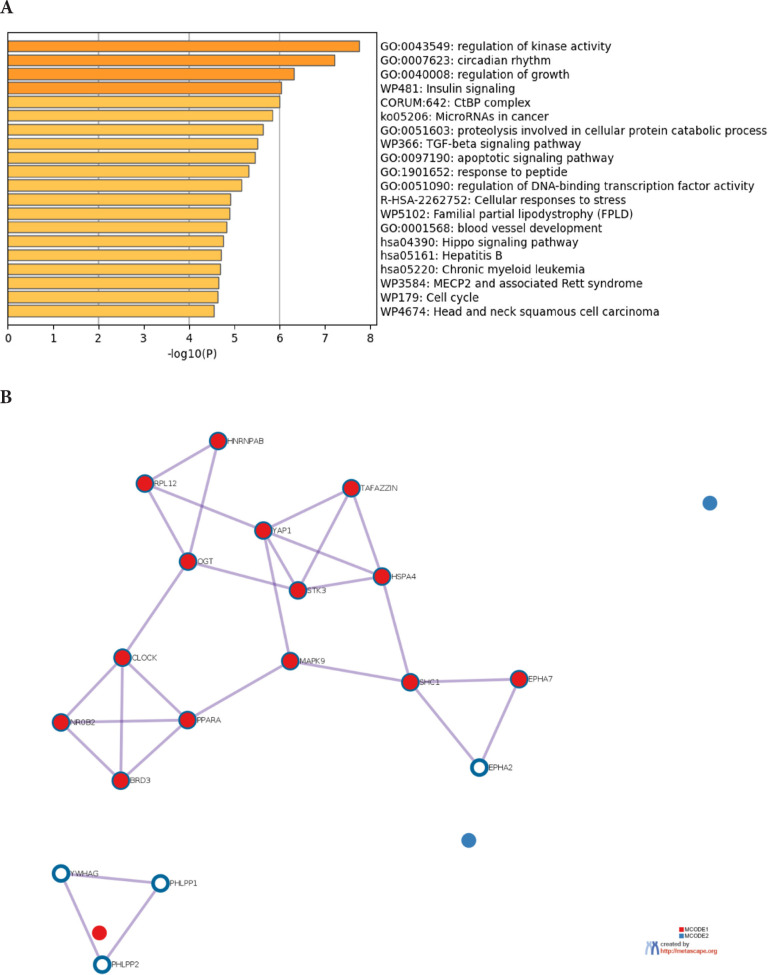
**Gene target prediction.** A) Functional enrichment analysis of target genes regulated by has-miR-141-3p sequence. B) Interaction network of the clusters detected by Metascape.

**Figure 5.**
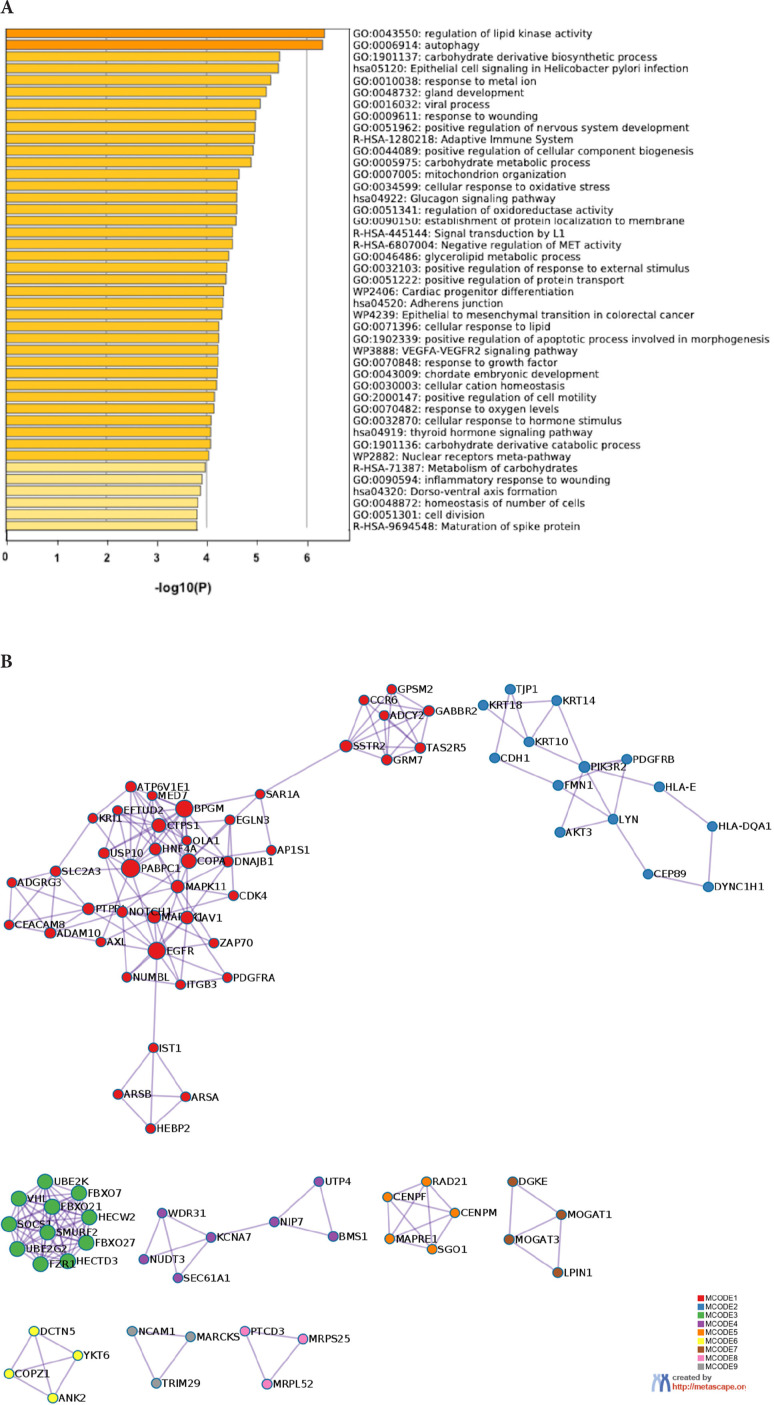
**Gene target prediction.** A) Functional enrichment analysis of target genes regulated by hasmiR-122-5p and has-miR-34c-5p sequence. B) Interaction network of the clusters detected by Metascape.

The prediction analysis of target genes regulated by has-miR-122-5p, has-miR-34c-5p, and hasmiR-141-3p was obtained using the miRTarBase software. A number of 773 genes were commonly regulated by the tested miRNAs. Among these genes, the chosen parameter (*P* value 0.05) selected 510 genes, which were subjected to GO enrichment and the PPI-Nets, via the METASCAPE tool. The target genes associated with the upregulated miRNA sequence (has-miR-141-3p) were closely associated with “regulation of kinase activity” (GO: 2790043549), “circadian rhythm” (GO: 0007623), “regulation of growth” (GO: 0040008) and “insulin signaling” (WP481) (Figure 4). On the other hand, the predicted target genes of the 2 down-regulated miRNA sequences (hasmiR-122-5p and has-miR-34c-5p) were significantly involved in the “regulation of lipid kinase activity” (GO: 0043550) and “Autophagy” (GO: 0006914) (Figure 5).

## DISCUSSION

The relatively high number of cases of unexplained male infertility represents a great challenge as many times it is not possible to detect any alteration by routine semen analysis, which mainly focuses on the count of sperm in the ejaculate and their morphology and motility. The association between urogenital infections and male infertility are known causes of infertility and has been studied with variable, inconclusive results.

*Ureaplasma parvum* has been recognized as a sexually transmitted infection that could impair human fertility. They are known to colonize the female and male reproductive tracts as commensals, and growing evidence has shown they are emerging sexually transmitted opportunistic pathogens able to cause asymptomatic chronic disorders affecting female and male fertility.

The bacteria *U. parvum* may have a negative effect on semen quality, which can lead to infertility by attaching to sperm and influencing vitality, motility, morphology, cellular integrity, or host factors and cellular interactions [35]. Even if the influence of some genital bacterial infections on sperm function and spermatogenesis has been highlighted, the role of these infections as the significant causes of male infertility is still arguable. Therefore, clinicians should consider these infections in the treatment of infertile couples.

In the past decade significant improvements have been made in our understanding of sperm cell biology and also the ability to diagnose and treat male infertility, considering the large number of genes involved in male infertility phenotypes of genetic origin. In the last decade, some articles described the presence of miRNAs in all human tissues, including the reproductive system [36, 37].

The miRNAs are essential molecules capable of affecting sperm quality and influencing different biological processes such as sperm maturation and capacitation [38]. As miRNAs are abundant in plasma, serum, and seminal plasma, it makes them appealing potential non-invasive biomarkers of sperm quality. Dysregulation of miRNAs is known to play a necessary role in male infertility. The presence of miRNAs in testis, epididymis, sperm cells, seminal plasma, and extracellular vesicles *(ie*, exosomes and microvesicles) and the known functions associated with these molecules suggest that deregulation of their expression may result in alterations in spermatogenesis and embryogenesis [39]. Spermatogenesis is a highly complex and regulated process that produces millions of sperm every day and miRNAs play an important role in spermatogenesis during the mitotic and meiotic phases of spermatogenesis through the regulation of targeted gene expression [40, 41]. Such perturbations could have the potential to give rise to various forms of infertility.

In particular, miR-141, miR-122, and miR-34 expression was found altered in sperms from zebrafish, affecting sperm motility and indicating a low-quality sperm [22, 23, 26]. Moreover, these miRNAs were altered also in spermatozoa from patients showing metabolic diseases or spermatogenic impairments [19, 24, 25].

Here we show for the first time that patients who had urogenital tract infected by UP had seminal fluids containing spermatozoa with altered expression of 3 miRNAs involved in sperm motility [42]: miR-122-5p, miR-34c-5p, miR-141-3p. These miRNAs were analyzed in spermatozoa and not in sperm cells, to detect their levels as cellular miRNAs and not as secreted ones. Notably, miR-122-5p and miR-34c-5p were significantly decreased in spermatozoa from infected patients versus non-infected while miR-141-3p was significantly increased in the spermatozoa from infected patients. Several studies already reported that alteration of a specific pattern of miRNA expression is associated with alterations of sperm motility known as astheno-zoospermic and oligo-asthenozoospermic conditions, so they have a potential to reflect the testicular condition. However, the possible modulation of host miRNAs by bacteria has not been so deeply explored until now that they could be considered as non-invasive and effective predictive biomarkers for complete spermatogenesis [30]. Only a single study on female patients reported modification of a specific endocervical miRNA pattern in patients with *Chlamydia trachomatis* while other studies on mouse models only hypothesized an association between different species of *Chlamydia* and specific host miRNAs in the female murine genital tract [43–47]. To our knowledge, no evidence reported a modulation of sperm miRNA by UP, and therefore this is a novel insight into exploring host miRNA modifications by bacteria. To deepen the issue in an attempt to clarify whether UP-induced miRNAs modifications influenced spermatozoa motility, the percentage of motile, progressively motile, not-progressively motile, and not-motile sperms has been analyzed here by recording significant differences among the seminal fluids considered infected against non-infected. Not-progressively motile and not-motile spermatozoa were higher in infected individuals compared to non-infected individuals [17, 25, 48]. Their motility was negatively correlated with miR-122-5p and miR-34c-5p expression, while positively associated with miR-141-3p expression. Therefore, infection of UP can precipitate sperm motility, and miRNAs are part of this action. In this context, although the relationship of sperm motility with changes in these 3 miRNAs was already reported [24], the data collected here relating to seminal fluids infected with UP are new.

From a mechanistic point of view, an attempt to explain how the UP infection modifies the miRNAs first, and then the sperm motility, is traced here by 2 pieces of evidence, i) the changes in spermatozoa epigenetics were paralleled by increased production of superoxide anion in infected sperms; ii) a post-hoc target gene analysis revealed an involvement of kinases activity in the UP-dependent effects [48–50]. The oxidative stress recorded in UP-infected sperms was so high compared to uninfected sperms that this factor is thought to have a key role in the observed changes in miRNAs [19, 30, 51]. This latter contention is supported by extensive literature showing that oxidative stress can regulate microRNA biogenesis and expression mainly through modulation of their biogenesis course, their transcription factors, and their epigenetic phenomena [52–54]. Worthy of note, miR-122-5p, miR-141-3p, and miR-34c-5p expression seem to be modulated by oxidative stress [49, 50, 52, 53, 55]. In the presence of infections, an imbalance of pro-oxidants and antioxidants occurs, in favor of oxidative stress. Supra-physiological levels of ROS affect sperm function and lead to infertility.

A post-hoc bioinformatics analysis was carried out to predict the pathways modulated by the miRNAs dysregulated in the differently motile spermatozoa [56–58]. A target gene prediction revealed that an essential role of these miRNAs resides in the regulation of lipid kinase activity, accounting for changes in the constitution of spermatozoa membrane lipids caused by UP [59]. It is noteworthy that PI3K, one of the 2 main lipid kinases along with SPHK1, can modulate sperm motility [60–63]. Alterations in the PI3K signaling pathway result in reduced motility [64, 65], increasing the rate of immobile and non-progressively motile spermatozoa. Further investigations are necessary however, aimed at a better understanding of the specific targets of the tested miRNAs deregulated by UP, for the selection of innovative diagnostic and therapeutic targets.

## CONCLUSION

In the present work it was shown that non-motile and non-motile progressive spermatozoa were higher in infected individuals than in non-infected individuals. Their motility was negatively correlated with the expression of miR-122-5p and miR-34c-5p, while positively associated with the expression of miR-141-3p. Although the relationship of sperm motility with changes in these 3 miRNAs has already been reported, this report provides evidence that the data collected here showed that UP-infected seminal fluids had sperm with increased superoxide anion, reduced expression of miR-122-5p, miR-34c-5, and increase of miR-141-3p compared to non-infected normospermic patients. Although our reported data are very valuable in a context where information on the role of these miRNAs in UP infections and their relationship with semen quality is limited, some limitations should be considered. The main limitation of the study is the small number of patients. Therefore, programs should be implemented with studies involving multiple hospitals to confirm these preliminary data.
